# Giant anteaters on the move: native habitat selection and behavioral responses to land use change

**DOI:** 10.1186/s40462-025-00616-8

**Published:** 2025-12-24

**Authors:** Ana Yoko Ykeuti Meiga, Arnaud Leonard Jean Desbiez, Rubem A. P. Dornas, Nina Attias, Aline Giroux, Denis Valle

**Affiliations:** 1 School of Natural Resources and Environment, University of Florida, Gainesville, FL USA; 2 Wild Animal Conservation Institute (ICAS), Campo Grande, Mato Grosso do Sul Brazil; 3 Royal Zoological Society of Scotland (RZSS), Murrayfield, Edinburgh, UK; 4 Road and Railroad Ecology Research Group (NERF-UFRGS), Federal University of Rio Grande do Sul, Porto Alegre, Rio Grande do Sul Brazil; 5 Center for Latin American Studies, University of Florida, Gainesville, FL USA; 6 School of Forest, Fisheries, and Geomatics Sciences, University of Florida, Gainesville, FL USA

**Keywords:** Animal behavior, Brazilian savanna, Cerrado, Conservation, Hidden Markov model, Landscape fragmentation, Mammals, Movement ecology, Telemetry

## Abstract

**Background:**

Landscape fragmentation and habitat loss are major drivers of global biodiversity decline. Understanding how animals adjust their behavior in response to these threats and adapt to human-altered environments is essential for developing effective conservation strategies. In this study, we apply Bayesian models to a unique movement dataset to explore how landscape transformation influences behavioral patterns in the giant anteater (*Myrmecophaga tridactyla*; Pilosa; Mammalia), a vulnerable species with limited physiological thermoregulatory capacity and strong behavioral responses to environmental changes.

**Methods:**

We used an extensive GPS tracking dataset from 41 giant anteaters in the Brazilian savanna—a biodiversity hotspot facing extensive landscape alteration due to land-use and land-cover change. We used the Time-Explicit habitat selection model to investigate giant anteater sex-based differences in habitat selection. Additionally, we evaluated how habitat types influence the species’ activity patterns using a nonparametric Bayesian Hidden Markov model.

**Results:**

Our study reveals that giant anteaters change their movement patterns in response to land use and land cover change. Individuals spent more time in and selected native habitats compared to human-modified habitats, regardless of sex. The time spent on, and selection for, native habitats are likely due to greater resources such as food, shelter, and protection from predation. Additionally, giant anteaters are more likely to rest in native habitats while being more active in human-disturbed areas.

**Conclusion:**

Our research reveals the negative impact of human-disturbed landscapes on animal movement. Consistent with our expectations, giant anteaters spend more time in native habitats and tend to avoid areas with high human disturbance, underscoring the urgent need for conservation efforts in degraded landscapes. Understanding how different land use and land cover classes influence animal habitat selection and activity patterns is essential for assessing the species’ adaptability and ecological requirements in human-modified environments. Our findings can guide the prioritization of critical areas for conservation and restoration, offering valuable insights for policymakers and supporting the effective management of this vulnerable species and the Brazilian savanna.

**Supplementary information:**

The online version contains supplementary material available at 10.1186/s40462-025-00616-8.

## Background

Human-driven land use changes, such as landscape fragmentation and habitat loss, are the main drivers of terrestrial biodiversity loss [1]. These processes lead to the reduction and isolation of natural areas, which become surrounded by a mosaic of land uses [2]. The impact of landscape change on wildlife depends on the species’ capacity to adapt to the new mosaic of habitats [3]. Animals may alter their behavior to cope with these new environments, which can negatively impact their survival rates and reproductive fitness [4]. Understanding the complex relationships between species and their habitats, particularly in regions undergoing rapid human-driven land transformation, is crucial for effective conservation [5].

Movement is a key behavior that allows animals to adjust to their environment and is essential in changing landscapes [6, 7]. In particular, animal responses to habitat change can be examined by habitat selection/avoidance [8, 9] and activity patterns [4, 9]. For instance, animals may move faster to avoid areas with limited resources and threats, while moving slowly in areas that offer better resources and conditions [10, 11]. Additionally, diel activity patterns are crucial for understanding energy and fitness trade-offs in animals. For example, while resting saves energy, it can limit essential activities like finding mates and caring for young, requiring a balance to maintain fitness [12]. Moreover, factors such as sex and individual personality also influence activity patterns [13]. For instance, males and females often have different reproductive roles, leading to distinct activity strategies [14]. Understanding these differences in activity patterns, especially when influenced by land use and land cover (LULC, hereafter abbreviated) changes, can provide crucial insights into animal adaptation to human-disturbed environments and their impact on individual fitness and survival [15].

Giant anteaters (*Myrmecophaga tridactyla*; Pilosa; Mammalia) have a low metabolic rate and low physiological thermoregulation capacity [16, 17], resulting in animals modulating activity period and seeking shelter in forests during extreme temperatures [18–20]. Their habitat use patterns are intrinsically related to thermoregulation, with animals typically foraging in open areas but relying on forests for rest and refuge [18–23]. Because the landscape plays a crucial role in the species’ thermoregulation and behavioral adaptations, giant anteaters serve as a valuable model species for understanding how landscape alterations may affect other mammalian species. The giant anteater is also of conservation concern, being listed as a vulnerable species by the IUCN Red List [24], threatened by habitat loss, poaching, roadkill, conflicts with domestic dogs, and wildfires [25, 26].

Despite improvements in data collection through GPS telemetry, much of the knowledge on the giant anteater’s movement is based on studies from protected or natural areas [19, 20, 27], particularly focused on the Pantanal region of Brazil [28]. Furthermore, many foundational studies were limited by small sample sizes or short monitoring durations [18, 29, 30]. While some studies have documented giant anteaters in agricultural fields and timber plantations [31, 32], these studies generally did not use GPS telemetry devices. Recent studies in modified landscapes used extensive data to explore giant anteaters’ behavior around roads [33] and their socio-spatial ecology [34]. However, significant gaps remain regarding giant anteater movement and behavior in human-modified landscapes. Notably, no studies have investigated giant anteater habitat selection in a degraded landscape in the Brazilian savanna and how different LULC classes influence their diel activity patterns.

This study investigates how changes in land use and land cover influence giant anteater movement patterns in the Brazilian savanna, a biodiversity hotspot where over half of the area has been converted to agriculture and other land uses, resulting in a highly fragmented and degraded landscape [31, 35]. We relied on an extensive telemetry study, tracking 41 individuals over a period of two years. Our goals were to: (1) investigate the time giant anteaters spend in different land uses and land cover classes; (2) analyze habitat selection or avoidance, focusing on differences in patterns by sex; and (3) evaluate how LULC classes influence giant anteater diel activity patterns. For the first goal, we hypothesized that giant anteaters would exhibit slower movement and spend more time in native habitats, indicating resource-rich habitats [9]. For the second goal, we expected that animals would select native environments due to greater resource availability in these areas, with females exhibiting a higher selection for native areas than males due to their extended parental care [36]. Females may select higher-quality habitats because of limited mobility during parenting, and native habitats likely offer better protection from predators, adverse weather, and human activities. Regarding the third goal, we predicted that giant anteaters would increase resting in native landscapes, mainly during warmer periods of the day, since native areas provide better resources, such as shelter and protection for resting [18–20].

## Methods

### Study site and data collection

**Fig. 1:**
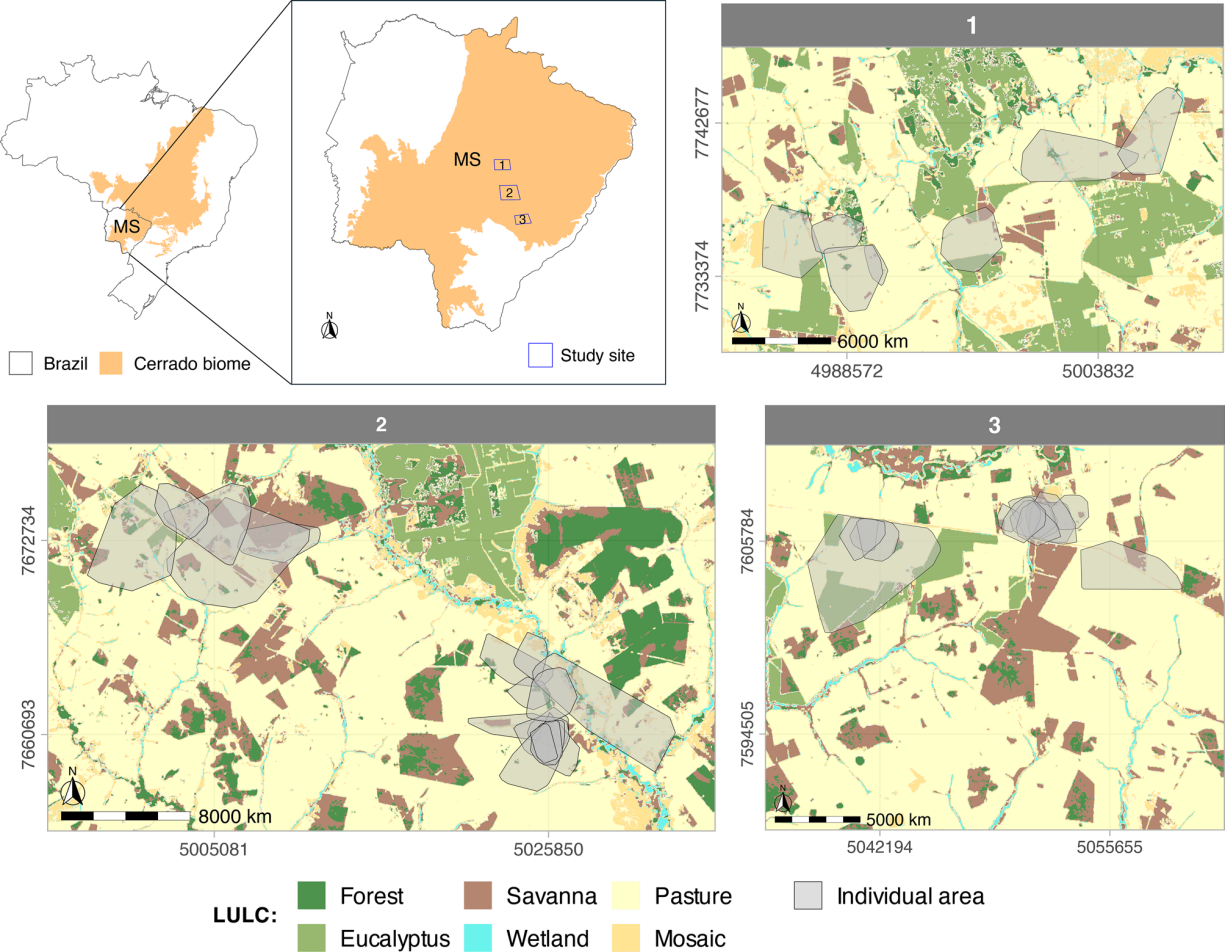
Maps of the study region. Map indicates the Cerrado biome in orange, the three study sites located in the state of Mato Grosso do Sul (MS), along with the predominant land use and land cover (LULC) classes for each site. The LULC classifications in the legend apply to all three areas and were derived from MapBiomas. Gray polygons depict the home range of each giant anteater monitored in the study sites, using the minimum convex polygon method

Our study was conducted in the Brazilian savanna (Cerrado) in Mato Grosso do Sul state, Brazil (Fig. 1). The Cerrado exhibits distinct seasonality, characterized by a wet period from October to March and a dry season from April to September (Koppen’s Aw), with the mean annual rainfall ranging from 1,000 to 1,500 mm [37]. The historical annual mean temperature fluctuates between 21º C and 32º C, with highs exceeding 40º C and lows reaching -3º C [38, 39]. The Cerrado is a global biodiversity hotspot, having the richest flora among the world’s savannas, with over 7000 species and a high level of endemism [40]. Particularly in Mato Grosso do Sul state, it features a mosaic of savanna, forest, and wetland habitats. However, the Cerrado has already lost 46% of its native vegetation, a result of the expansion of extensive cattle ranching and agricultural activities, including soybean, corn, and sugarcane cultivation, as well as forest plantations like *Eucalyptus* [41, 42]. As shown in Fig. 1, the land use and cover at our study site is predominantly pasture, with remnants of native forest and savanna, and areas covered by *Eucalyptus* and crop plantations [33].

We used telemetry data collected in 2017 and 2018 with GPS TGW-4570–4 Iridium. GPS locations of 41 giant anteaters were recorded every 20 minutes across different land uses in three sites (Fig. 1). Collared giant anteaters were monitored every two weeks for general health inspections and recaptured after approximately 12 months to download data. Capture and recapture methods followed the Guidelines of the American Society of Mammologists for use of wild mammals in research [43], described in detail in [44]. The procedures were authorized by the Brazilian Ministry of the Environment (MMA), Instituto Chico Mendes de Conservação da Biodiversidade (ICMBio), under license 53.798–7.

### Movement data processing

Our dataset originally encompassed 847,671 GPS locations from 41 giant anteaters (females *n* = 21; males *n* = 20). We preprocessed the data from all individuals to remove location errors from GPS. To remove location errors, we first eliminated paths that suggested unrealistic speeds by excluding step lengths that were too large for the correspondent time interval, determined as the top 1% of step lengths. Additionally, we excluded steps with excessively long time intervals ( > 60 min) due to the missing of 3 or more GPS fixes. After these preprocessing steps, our dataset had 795,919 observations from 41 animals, corresponding to 93.9% of the original data. GPS data is available on MoveBank Data Repository [45], study number 1574830796, and all the procedures were conducted usin R [46].

### Land use and land cover (LULC)

To characterize the land use and land cover of the study region, we relied on the LULC product from MapBiomas Collection 7. MapBiomas is a peer-reviewed initiative that provides annual, high-resolution (30 m) land use and land cover maps for Brazil, based on the Landsat satellite archive [47, 48]. We relied on the 2018 LULC product to characterize the landscape for our study period.

To extract the LULC information at each point of the giant anteater individual tracks, we first generated a line segment for each pair of consecutive GPS points (i.e., hereafter referred to as a step) and created a 30 m buffer around this line. Then, we extracted raster information within this buffer zone and calculated the LULC proportions [9]. We eliminated LULC classes that were consistently rare across all animals (i.e., grassland, river and lake, sugar cane, soybean, other temporary crops, and other non-vegetated areas), defined as classes that were consistently less than 10% of the buffer area for all individuals. As a result, only the more dominant LULC classes were kept in our analysis (i.e., forest, wetland, savanna, forest plantation (*Eucalyptus*), pasture, and mosaic of uses). The land use denominated “mosaic of uses” is classified by MapBiomas as a combination of agricultural use without differentiating between pasture and agriculture.

### Data analysis

#### Time-Explicit habitat selection (TEHS) model

To address goals 1 and 2, we used the Time-Explicit habitat selection (TEHS) model [9] to understand how landscape composition influences animal movement decisions. We chose to use the TEHS model in our analysis, instead of other conventional methods like integrated step selection analysis (iSSA [10]), because it provides not only information on habitat selection but also crucial additional insight into the factors that influence the time spent in each habitat type. Rather than considering habitat selection based only on the start and end points (steps), the TEHS model considers the entire path between the animal’s starting and ending points. For instance, animals often move faster to avoid areas with limited resources or higher threats, while moving more slowly in regions that offer better resources and conditions [10, 11]. Therefore, integrating the time animals take to move through the habitat, along with the strength of habitat selection, can provide a more nuanced understanding of animal activities and their interactions with their environment. This is particularly relevant for understanding the impact of landscape degradation and fragmentation on animal behavior [9]. The TEHS model is comprised of two components: a time submodel and a habitat selection submodel. The time submodel considers how different factors (covariates) influence the time it takes for the animal to move through the landscape, whereas the habitat selection submodel assesses how the animal selects one habitat type over another (e.g., greater selection for forest compared to pasture) [9].

The TEHS model simultaneously estimates both movement and resource selection for each step [9, 10]. Before implementing the TEHS, we pre-processed the data to replace all GPS fixes with zero distances with the shortest step length present in our dataset (1 meter), given that the time submodel requires distances greater than zero. Our time submodel incorporates different LULCs as covariates and pasture as the baseline since pasture is the predominant LULC in our study site. The specific LULC classes used as covariates were forest, savanna, wetland, *Eucalyptus* plantation, and mosaic of uses. The time submodel only includes linear terms for LULC covariates (i.e., no interactions or non-linear effects were included), which assumes that the time taken for a particular step is related to distance (i.e., step length) and LULC characteristics along that path [9].

In the habitat selection submodel, we established potential paths for the animal by delineating four alternative steps identical in length to the observed step, each with a different direction (east, west, north, and south) from the initial point of the step, as described in [9]. Substantially increasing the number of steps would make the model take much longer to fit, an important problem given that separate models were fitted for each of the 41 individuals. As with the time submodel, we used pasture as a baseline in our analysis because pasture was the predominant LULC in our study site, and the same set of covariates was used (i.e., proportion of forest, savanna, wetland, *Eucalyptus* plantation, and mosaic of uses). Because pasture was the baseline, it was excluded from our set of LULC covariates [49]. The habitat selection submodel considers distance (step lengths), travel time, and landscape characteristics [9]. We fit these submodels in a Bayesian framework using JAGS [50]. For equation details, see Additional file 1: Appendix 1.

Because one must be careful when interpreting regression results if proportion covariates are present [49], we focused on comparing different LULC classes by reporting ratios. For the time submodel, we calculated the ratio of the time taken to traverse one type versus another type of LULC class. As a result, a ratio of time greater than 1 indicates increased time spent in a particular LULC (slower movements) when compared to the other LULC. In contrast, a time ratio lower than 1 denotes less time spent in a particular LULC (faster movements) when compared to the other LULC. For the habitat selection submodel, an odds ratio (OR) greater than one indicates selection, while an OR less than one indicates avoidance [51, 52]. We analyzed the odds ratio by giant anteaters’ sex through post hoc stratification to determine if there was a difference in habitat selection between males and females.

#### Hidden Markov model – activity patterns

For goal 3, we identified giant anteater activity and resting periods using a nonparametric Bayesian Hidden Markov model (HMM). The advantage of this approach is that it does not rely on parametric distributions to model step lengths and turning angles (e.g., gamma and von Mises distributions). Rather, it discretizes these variables and models them with a categorical distribution. We discretized step length, creating eight distinct bins with breakpoints given by 0, 30, 60, 90, 120, 150, 180, 210, and 512 meters. The value of 512 corresponds to the 99.9th percentile of step length. We discretized turning angles by creating 8 bins of equal width between -π and π. We fit this HMM, independently of the TEHS model, by running a customized Markov Chain Monte Carlo (MCMC) algorithm for 1,000 iterations, discarding the first 500 as burn-in. We confirmed model convergence by visually inspecting trace plots of the likelihood. We pre-specified two possible behavioral states (i.e., resting and active) because it can be increasingly challenging to interpret behavioral states if more states are estimated, and it is virtually impossible to ensure that these additional behavioral states are the same for different individuals. Finally, we believe that these two behavioral states are biologically meaningful, as giant anteaters tend to rest for long periods and forage with rapid feeding stops lasting under a minute to avoid bites and chemical secretions from ants and termites [53, 54].

To display the diel activity patterns based on the HMM results, we discretized the hours of the day in 11 bins, each representing 2 hours (from 12 to 2 am, 2–4 am, 4–6 am, 6–8 am, 8–10 am, 10–12 pm, 12–2 pm, 2–4 pm, 4–6 pm, 6–8 pm, 8–10 pm, and 10 pm–12 am). This allowed us to calculate the proportion of movements classified as “active” and “resting” as a function of time of day. Specifically, we calculated the proportion of resting behavior by LULC class and hour, allowing us to visualize the diel activity patterns based on the HMM results.

## Results

### TEHS model

**Fig. 2:**
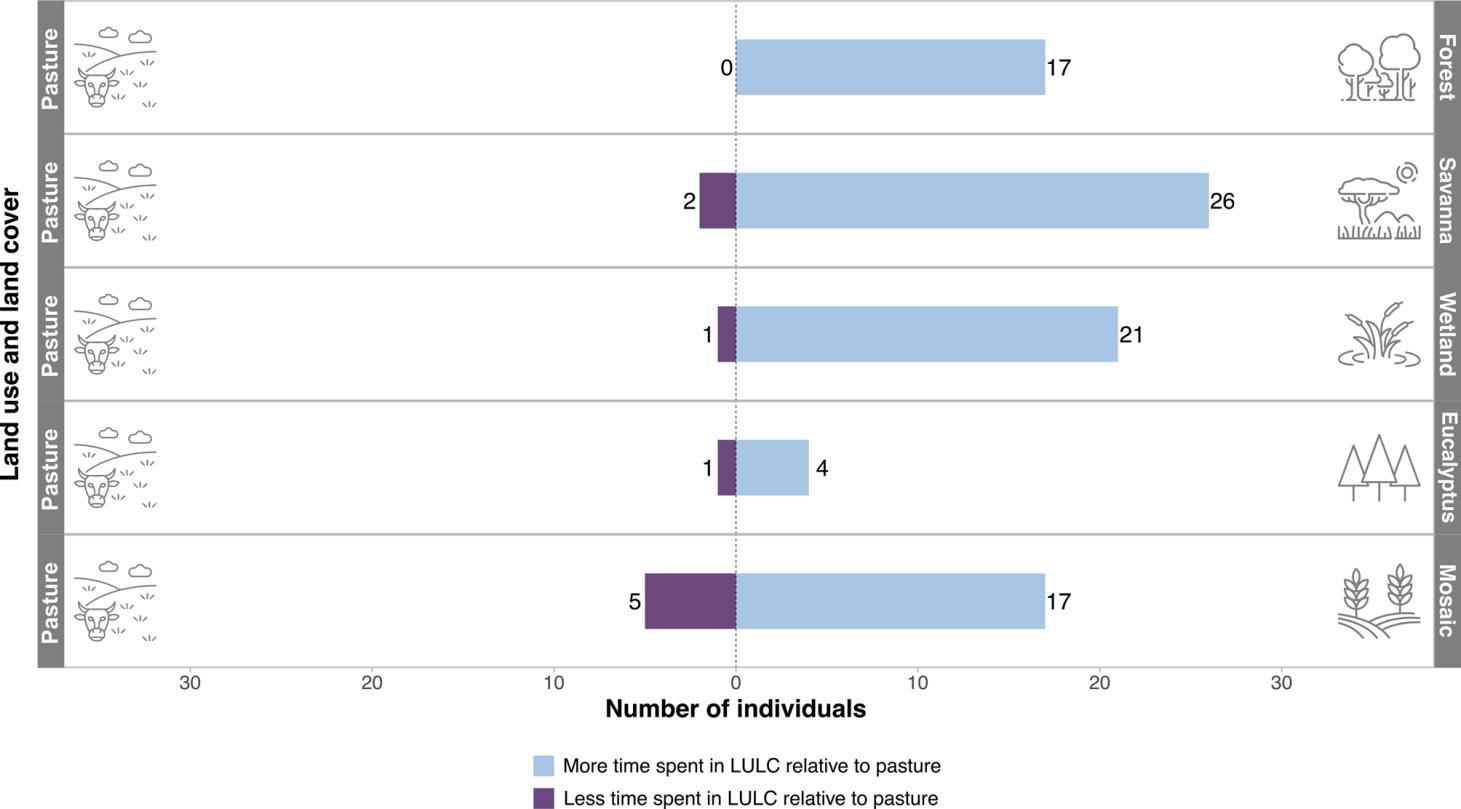
Results from the TEHS time submodel indicating that most individuals spend more time crossing other LULC classes when compared to pasture. Blue bar size represents the number of individuals that statistically spent more time in other LULC relative to pasture. Purple bar size represents the number of individuals that statistically spent less time in other LULC classes compared to pasture

The time submodel outcomes indicated that most giant anteaters moved faster in pastures compared to all other LULC classes (Fig. 2). In our analysis, all individuals spent more time in forests than in pastures. Additionally, 93% of the individuals spent more time in savannas compared to pastures, and 91% spent more time in wetlands than in pastures. Regarding the *Eucalyptus* plantation, 80% of giant anteaters spent more time in *Eucalyptus* plantations compared to pastures, and 77% of them spent more time in the mosaic of uses rather than in pastures.

**Fig. 3:**
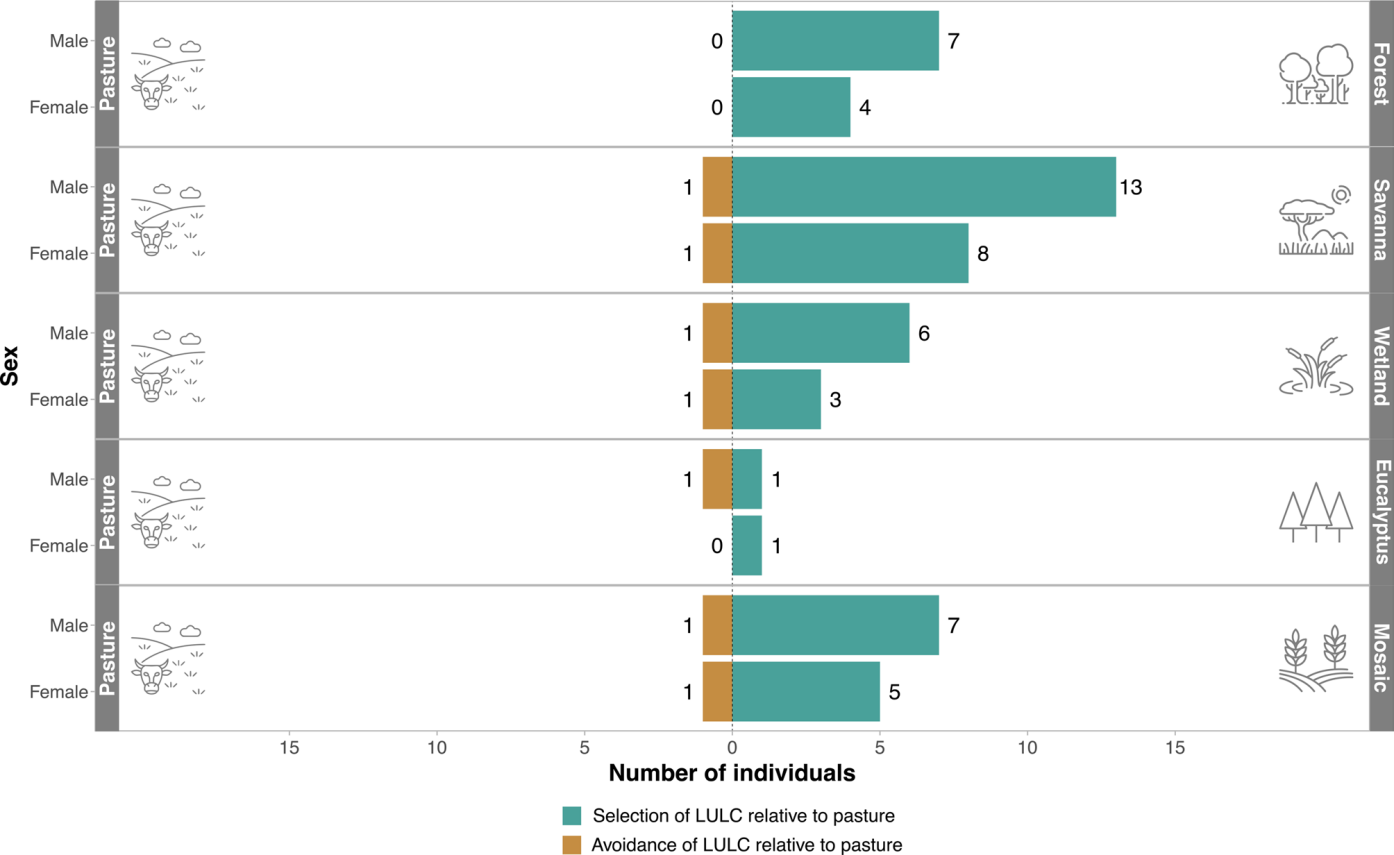
Sex-stratified results from the TEHS habitat selection submodel indicating greater selection of other LULC classes when compared to pasture. Green bars indicate the number of individuals that statistically selected a target LULC class when compared to pasture. Brown bars indicate the number of individuals that statistically avoided a target LULC class compared to pasture

In relation to the habitat selection submodel results, our findings consistently showed that giant anteaters select native habitats compared to pasture. This selection was particularly evident for native forests (100%) and savannas (91%). On the other hand, our results did not reveal any distinct pattern in habitat selection based on sex (Fig. 3).

### Hidden Markov model – activity patterns

**Fig. 4:**
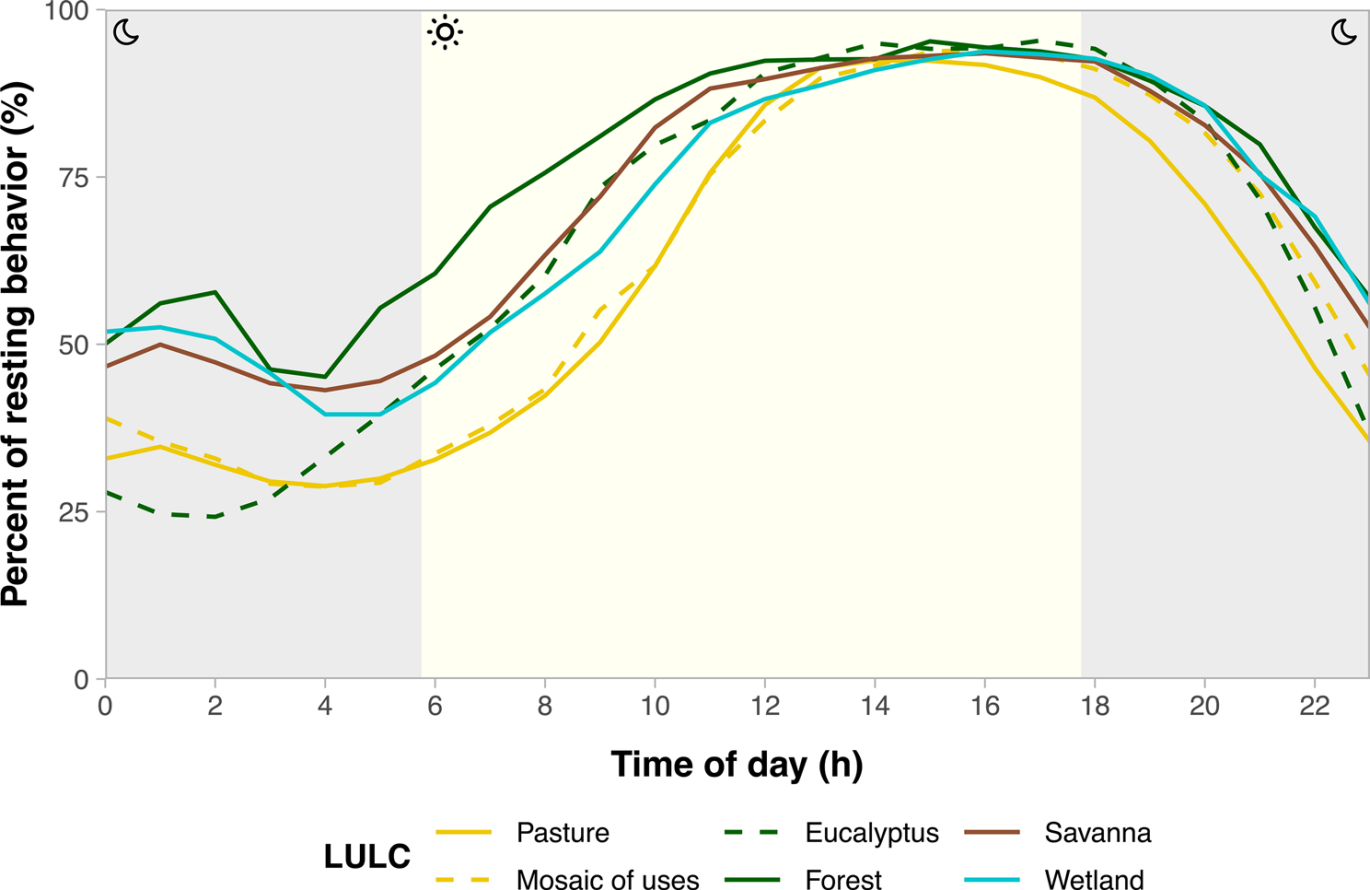
Giant anteater resting patterns across different hours of the day and LULC classes. The estimated proportion of time spent resting (y-axis) is based on the Hidden Markov model classification of observations into resting and active classes. Colors and line types indicate distinct LULC classes.

Based on the distribution of step lengths and turning angles, our analysis of HMM distinguished “active” and “resting” states for each individual (Additional file 2: Appendix 2). When calculating the proportion of resting throughout the day, we observed that giant anteaters tend to rest between 12 h and 18 h, regardless of habitat type. The only exception is in pasture, where giant anteaters are less likely to rest compared to other LULC classes (Fig. 4). During the colder hours of the night and in the early morning, there was a higher variation in resting behavior among LULC classes. Giant anteaters rested more frequently in less disturbed habitats, such as savanna, forest, and wetland, whereas the proportion of resting was lower in pastures and mosaic of uses compared to other LULCs (Fig. 4). Moreover, giant anteaters exhibit different resting patterns in *Eucalyptus* plantations compared to native forests. Specifically, from midnight to 4 h, the proportion of resting was higher in forests than in *Eucalyptus* plantations (Fig. 4). On the other hand, giant anteaters’ diel activity patterns were similar in the pasture and the mosaic of uses. The active patterns of giant anteaters show that the species is more active during nocturnal periods, especially in human-disturbed environments such as pasture, mosaic of uses, and *Eucalyptus* plantation (Additional file 3: Appendix 3).

## Discussion

Understanding the intricate relationships between species and their habitats is crucial for effective conservation, particularly in landscapes undergoing rapid anthropogenic transformation [5]. In this study, we investigated how changes in land use and land cover classes influence the habitat selection and activity patterns of giant anteaters in a region under significant human-driven landscape alterations. We found evidence supporting our hypothesis that giant anteaters spend more time in, and tend to select for, native habitats. However, contrary to our expectations, our results indicated that female anteaters did not exhibit stronger selection for native habitats compared to males. Finally, as anticipated, we observed that individuals were more likely to rest in native environments, while being more active in human-disturbed areas like mosaics of uses and pastures. These findings provide new insights into how human-driven landscape changes influence animal movement and behavior.

As expected, our findings reveal that giant anteaters spend more time in, and tend to select for, native habitats, likely because these areas offer greater resources such as shelter, food, and protection from predators. Similar observations were made in natural environments, where giant anteaters seek shelter in forested areas to cope with extreme temperatures [19]. Additionally, ants and termites, the primary food source for giant anteaters, tend to be more abundant in native habitats in the Cerrado [55] while human activity tends to reduce ant richness and abundance [56, 57]. Moreover, analyzing both the time taken to traverse an area and habitat selection strength suggests that slower movement in a selected habitat likely indicates high-quality resources [9]. It is important to note that, had we only analyzed the time to traverse a landscape, slower movement could have been the result of obstacles to movement [58]. Thus, assessing both time and selection strength helps clarify different motivations for movement. Interestingly, our TEHS model also revealed that anteaters move faster and avoid pastures, indicating that pastures are permeable but perceived as risky habitats [9]. Giant anteaters’ pattern of selecting native habitats aligns with previous findings using conventional monitoring methods (e.g., traces, scat detection) from well-preserved Cerrado regions, reinforcing the importance of intact ecosystems [31]. Although giant anteaters use a wide variety of habitats, from open areas to forests [22, 53, 59–62], our findings underscore the importance of native forest and savanna for the species’ survival.

We hypothesized that females would exhibit stronger selection for native habitats due to limited mobility during parenting [14] and the likely benefits these environments offer against predators, extreme weather, and human activities. This behavior pattern has been observed in other Xenarthra species [63] and carnivores [64]. However, our TEHS results indicated that both males and females similarly select native habitats, suggesting that LULC classes can significantly impact the survival of both sexes. One possible explanation could be the similar body sizes of males and females in our study, as indicated in a previous study using the same dataset (males 32.89 ± 3.35 kg; females 32.77 ± 4.02 kg) (median ± SD) [44]. Alternatively, the absence of territoriality, mate-guarding, or other similar social behaviors suggests that giant anteaters are unlikely to show social dominance in habitat selection [34]. We acknowledge that seasonality was not explicitly modeled, and additional factors might influence the lack of sex-based differences in habitat selection. For instance, analyzing data from female individuals across the entire two-year monitoring period without accounting for their reproductive status may obscure sex-specific patterns in habitat selection. Female giant anteaters exhibit prolonged parental care, with offspring remaining on their mother’s back for six to nine months [36, 65]. The interval between pregnancies is approximately nine months, suggesting that the females in our study site potentially had more than one pup in this time period [66]. Future research should focus on critical periods for females (e.g., pregnancy or raising offspring) to better understand the influence of sex on giant anteater habitat selection. We also acknowledge that the TEHS model does not account for temporal or spatial correlation. However, similar to individual-level random effects, having a separate model for each individual helps to alleviate part of the temporal autocorrelation [9].

Despite the increasing availability of movement data, detailed information linking animal movement to diel activity patterns across different habitat types remains limited. Our study helps to fill a gap in understanding how different LULC classes influence the activity patterns of giant anteaters. Based on the results from the HMM, we found that individuals were more likely to rest in native habitats such as savannas, forests, and wetlands while being more active in human-disturbed environments like mosaics of uses and pastures. Resting is crucial for giant anteaters’ survival as it compensates for their low body heat production, thereby assisting the animal in their thermoregulation [16, 21, 67]. This behavioral plasticity aligns with patterns observed in other species, such as roe deer, one of the few documented cases showing the shift in activity patterns in response to agricultural land use [68]. Our findings also revealed that the proportion of resting was higher in native forests compared to *Eucalyptus* plantations. In contrast, another study found that giant anteaters favored timber plantations (*Acacia mangium*) over open savanna [32]. An important difference between our study and [32] is that we relied on GPS data collected over two years, while their findings were based on direct daylight observations of animal densities. Additionally, our results focused on *Eucalyptus* plantations, which have a lower biomass of soil macrofauna compared to *Acacia* and other pine species [69]. We recognize that the number of individuals observed in the *Eucalyptus* plantations is small. Thus, these results should be interpreted with caution. Further research in areas with *Eucalyptus* plantations could clarify their potential as high-quality habitats and inform timber plantation management strategies. Similarly, given the expansion of crop plantations in the Cerrado, it will become increasingly important to deepen our understanding of the effect of these anthropogenic environments on wildlife.

A recent study predicts significant changes in giant anteater spatial distribution due to climate change, highlighting the urgent need for conservation planning for this species [70]. As species’ habitat shrinks and climate change persists, competition for resources within smaller patches may increase, leading to reduced population sizes and genetic challenges [70]. Additionally, climate change poses significant survival challenges for species with low thermoregulation capacity, including armadillos and small anteaters in the Neotropics, as well as pangolins in Southeast Asia [71, 72]. In particular, the observation of albino giant anteaters in our study region raises concerns about genetic diversity [73], as albinism is often associated with small population sizes and increased inbreeding [74, 75]. Indeed, previous studies have documented low genetic diversity and high inbreeding levels in protected areas in the Cerrado [76]. One study found that the giant anteater population has moderate genetic diversity in our study region, but it also indicated that the population has already experienced a recent bottleneck, potentially due to increased human activities in the area [77]. Finally, the ecological importance of the Cerrado for the conservation of xenarthrans (Mammalia) has been emphasized, highlighting the need to prioritize areas that are currently suitable and may remain suitable in the future [78].

Our study was conducted in a landscape undergoing intense habitat loss and fragmentation, primarily driven by land-use changes such as pasture expansion for cattle ranching and large-scale soybean cultivation [35]. While this pattern is especially pronounced in the Brazilian Cerrado, it mirrors a global trend of natural habitat reduction and isolation within increasingly heterogeneous land-use mosaics [1, 2]. Despite these land use alterations, our findings show that giant anteaters persist in human-modified environments, although with behavioral adjustments. This suggests that while giant anteaters can adapt to human-disturbed landscapes, they still select areas with less anthropogenic influence when available. We argue that maintaining a mosaic of habitats (including native habitat) within anthropogenic areas is critical. In Brazil, the Forest Code requires that private rural landowners preserve a portion of natural vegetation and prohibits clearing in sensitive areas like riparian forests and hilltops. However, the Cerrado has fewer legal protections than the Amazon, requiring only 20% of properties to remain as a “legal reserve,” compared to 80% in the Amazon [35, 79]. Several studies have highlighted the importance of private properties in conservation and the need to improve compliance with these laws to restore protections for native vegetation [55, 80]. In particular, restoration programs on private properties lacking native vegetation can support threatened species [81]. Our study can help identify key habitat for conservation and restoration, providing important insights for policymakers and supporting effective management of giant anteaters and other species within the Cerrado.

Given the anteater’s strong responses to environmental variations, it may act as a model for broader biodiversity responses. As human-altered environments continue to expand, understanding how these changes influence animal movement, especially across different environmental conditions and different species, becomes increasingly important for predicting responses and informing conservation efforts [82]. Although the availability of biologging data has grown for certain species, many habitats and taxa remain underrepresented, limiting our ability to draw broad ecological inferences [82]. Recent research also highlights the importance of long-term datasets and sufficient sample sizes to support robust ecological conclusions [82]. These efforts are critical for refining our knowledge of species’ responses to environmental change and for supporting evidence-based management in dynamic and threatened ecosystems. By contributing new insights based on high-resolution movement data from a vulnerable mammal in the heavily degraded Cerrado, recognized as the largest savanna in the world [35], our study helps fill key knowledge gaps and underscores the urgent need for conservation strategies that account for behavioral adaptation in human-modified landscapes.

## Conclusion

This study provides valuable insights into how a vulnerable mammal adapts its movement patterns in response to different land use types. Our findings reveal that giant anteaters adjust their behavior by spending more time in native environments and strongly selecting forest and savanna habitats, even in landscapes undergoing extensive fragmentation and habitat loss, such as our study site. These results are essential for identifying priority habitats and informing targeted preservation and restoration initiatives within the Cerrado region. By revealing how different LULC classes influence diel activity patterns, our study sheds light on the species’ behavioral flexibility and needs in human-altered environments. This knowledge enhances our understanding of the challenges giant anteaters face in degraded landscapes and supports the development of more effective, data-driven conservation strategies. Moreover, given the species’ strong behavioral responses to environmental variation, giant anteaters serve as a valuable model for anticipating how other wildlife may respond to ongoing landscape transformation. Our findings underscore the urgency of conserving and restoring native habitats in rapidly changing ecosystems to ensure the long-term persistence of threatened species in the face of global change.

## Electronic supplementary material

Below is the link to the electronic supplementary material.

Supplementary Material 1

Supplementary Material 2

Supplementary Material 3

## Data Availability

Movement data for the giant anteaters analyzed during the current study are available for visualization in Movebank Data Repository (www.movebank.org), study number 1574830796, and study name “Giant Anteaters and Roads (Myrmecophaga tridactyla)”. Data downloads are available upon email requests. The LULC class data are freely available at Mapbiomas (https://mapbiomas.org/en/download).

## Abbreviations

HMM: Hidden Markov model
GPS: Global positioning system
iSSA: Integrated step selection analysis
JAGS: Just another Gibbs sampler
LULC: Land use and land cover
MCMC: Markov Chain Monte Carlo
OR: Odds ratio
TEHS: Time-Explicit habitat selection model
