# Supplementary material for: Giant anteaters on the move: native habitat selection and behavioral responses to land use change
Source: Mov Ecol. 2025 Dec 24;14:2. doi: 10.1186/s40462-025-00616-8 (PMC12805733; doi:10.1186/s40462-025-00616-8)
Supplement: Supplementary file 1 — Supplementary Material 1 [file 40462_2025_616_MOESM1_ESM.docx]

# Appendix 1: Time-Explicit Habitat Selection Model equations.

Time submodel:

Before implementing the TEHS, we pre-processed the data to replace all GPS fixes with zero distances with the shortest step length present in our dataset (1 meter), given that the time submodel requires distances greater than zero. Our time submodel incorporates different LULCs as covariates and pasture as the baseline since pasture is the main LULC in our study site. The time submodel is a Gamma regression, given by the equation below:

$$\Delta t_{j} \sim Gamma {(a}_{j}, b)$$

where $\Delta t_{j}$ represents the time taken for step $j$, and where the mean of $\Delta t_{.j}$is a function of step-length and landscape characteristics along the path traversed (assuming a straight line) by the animal.

Habitat selection submodel:

In the habitat selection submodel, we established potential paths for the animal by delineating four alternative steps identical in length to the observed step, each with a different direction (east, west, north, and south) from the initial point of the step. Our covariates consisted of the proportion of different LULCs within a 30 m buffer along the observed and alternative steps. We used pasture as a baseline in our analysis because pasture was the predominant LULC in our study sites, and it was therefore excluded from our set of LULC covariates. The habitat selection submodel uses a conditional logistic regression, given by the equation below:

$$L\left( \beta\right)= \prod_{i} \frac{pmov_{0i} \times exp\left( \boldsymbol{x}_{\boldsymbol{0}\boldsymbol{i}}^{\boldsymbol{T}}\boldsymbol{\beta} \right)}{pmov_{0i} \times exp\left( \boldsymbol{x}_{\boldsymbol{0}\boldsymbol{i}}^{\boldsymbol{T}}\boldsymbol{\beta} \right)+\ldots+ pmov_{4i} \times exp\left( \boldsymbol{x}_{\boldsymbol{4}\boldsymbol{i}}^{\boldsymbol{T}}\boldsymbol{\beta} \right)}$$

where $\boldsymbol{x}_{\boldsymbol{0}\boldsymbol{i}}^{\boldsymbol{T}}$ is a vector that contains the covariates for the realized step $i$ while $\boldsymbol{x}_{\boldsymbol{1}\boldsymbol{i}}^{\boldsymbol{T}}$, … , $\boldsymbol{x}_{\boldsymbol{4}\boldsymbol{i}}^{\boldsymbol{T}}$ contain the covariates (land use and land cover) for the matched potential steps 1, ..., 4, respectively. In this expression, $pmov_{0i}$ is the likelihood (based on the Gamma regression described above) of the realized step $i$ whereas $pmov_{1i}$, …, $pmov_{4i}$ are the probabilities for the matched potential steps 1, ... , 4, respectively.
