# Supplementary material for: Giant anteaters on the move: native habitat selection and behavioral responses to land use change
Source: Mov Ecol. 2025 Dec 24;14:2. doi: 10.1186/s40462-025-00616-8 (PMC12805733; doi:10.1186/s40462-025-00616-8)
Supplement: Supplementary file 2 — Supplementary Material 2 [file 40462_2025_616_MOESM2_ESM.docx]

# Appendix 2: Step length and turning angle distribution

Our Hidden Markov Model (HMM) estimated two distinct behavioral states based on the distribution of step lengths and turning angles. Faster step lengths and more direct movement were labeled as “active”, and slower movements (lower step lengths) with tortuous movements (higher turning angles bins) were labeled as “resting”. Behavioral states were identified for each giant anteater (41 individuals), and we present an example for two individuals (Alexander and Annie).

| 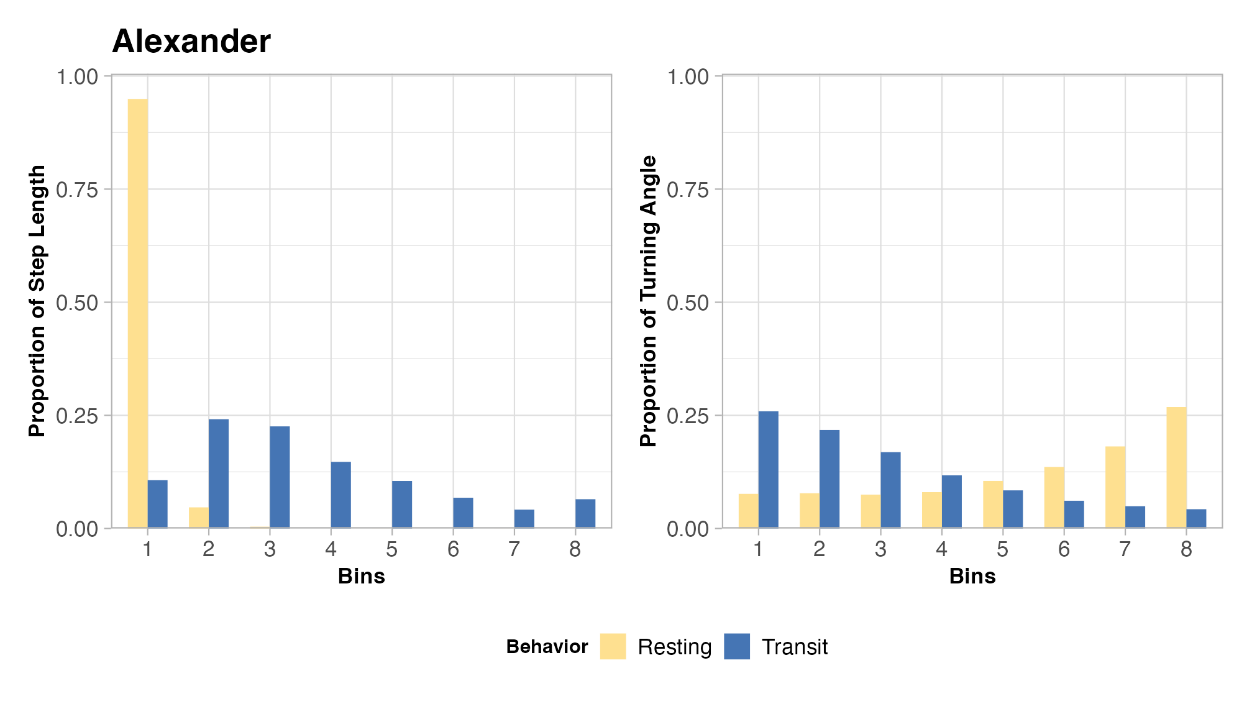 |
| --- |
| 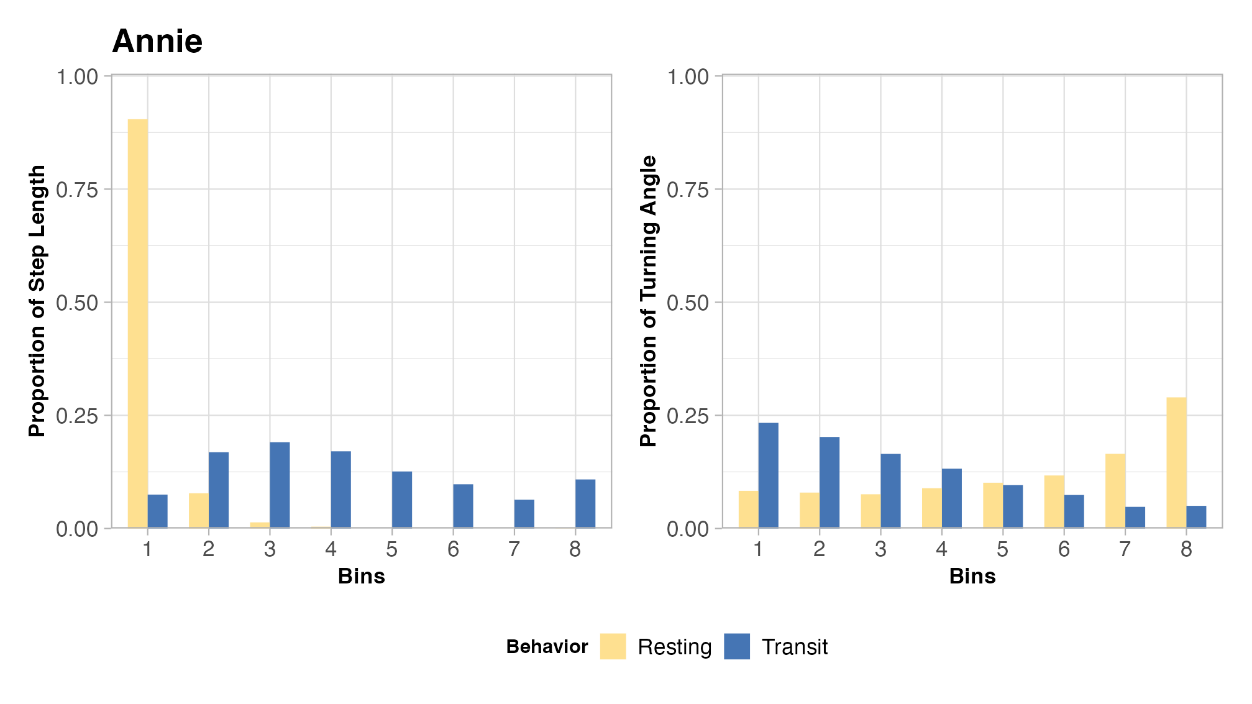 |
| Figure A.1. Example of behavioral state distribution for two individuals from HHM. Step length and turning angles were discretized in bins. Step lengths with higher bin values indicate faster movements, whereas higher bins for turning angles indicate more tortuous movement. Step length and turning angles were provided by GPS device. |
