# Supplementary material for: Giant anteaters on the move: native habitat selection and behavioral responses to land use change
Source: Mov Ecol. 2025 Dec 24;14:2. doi: 10.1186/s40462-025-00616-8 (PMC12805733; doi:10.1186/s40462-025-00616-8)
Supplement: Supplementary file 3 — Supplementary Material 3 [file 40462_2025_616_MOESM3_ESM.docx]

# Appendix 3: Active patterns of giant anteaters.

Based on our HMM results, we present the activity patterns of giant anteaters, which are the inverse of their resting patterns. Giant anteaters are more active during nocturnal periods, especially in human-disturbed environments such as pasture, mosaic of uses, and *Eucalyptus* plantation.

|  |
| --- |
| Figure A.2. Giant anteater active patterns across different hours of the day and LULC classes. The estimates depicted correspond to the proportion of time spent active. Colors and line types indicate distinct LULC classes. |
